# Supplementary material for: Modulation of α-Mannosidase 8 by Antarctic Endophytic Fungi in Strawberry Plants Under Heat Waves and Water Deficit Stress
Source: Int J Mol Sci. 2025 Dec 1;26(23):11650. doi: 10.3390/ijms262311650 (PMC12691858; doi:10.3390/ijms262311650)
Supplement: Supplementary file 1 [file ijms-26-11650-s001.zip › ijms-3995991-supplementary.pdf]

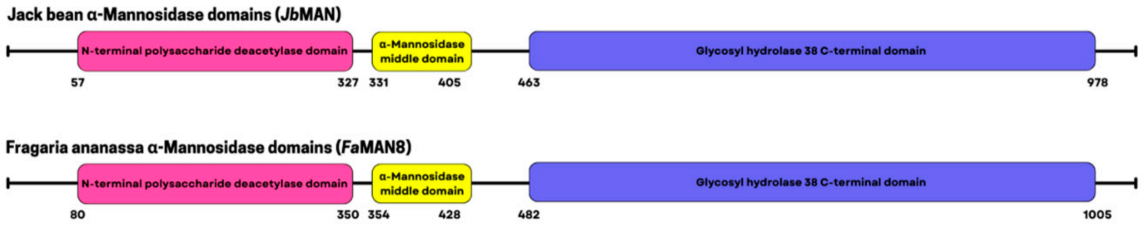

Figure S1. Comparison of domain architecture between FaMAN8 and its template JbMAN. Both proteins share a similar domain organization, including the polysaccharide deacetylase-like domain, the α-mannosidase middle domain, and the GH38 catalytic domain, supporting structural and functional conservation.
